# A randomized, double-blind, placebo-controlled pilot study of a probiotic in emotional symptoms of chronic fatigue syndrome

**DOI:** 10.1186/1757-4749-1-6

**Published:** 2009-03-19

**Authors:** A Venket Rao, Alison C Bested, Tracey M Beaulne, Martin A Katzman, Christina Iorio, John M Berardi, Alan C Logan

**Affiliations:** 1 Department of Nutritional Sciences, University of Toronto, 50 College Street, Toronto, Ontario M5S 3E2, Canada; 2 Environmental Health Clinic, Women's College Hospital, 76 Grenville Street, Toronto, Ontario M5S 1B2, Canada; 3 Integrative Care Centre of Toronto, 3600 Ellesmere Road, Unit 4, Toronto, Ontario M1C 4Y8, Canada; 4 Department of Psychiatry, University of Toronto, 250 College Street, Toronto, Ontario M5T 1R8, Canada; 5 START Clinic for Mood and Anxiety Disorders, 790 Bay Street, Toronto, Ontario M5G 1N8, Canada; 6 Precision Nutrition, 1665 Gregory Road, St Catharines, Ontario L2R6P9, Canada

## Abstract

Chronic fatigue syndrome (CFS) is complex illness of unknown etiology. Among the broad range of symptoms, many patients report disturbances in the emotional realm, the most frequent of which is anxiety. Research shows that patients with CFS and other so-called functional somatic disorders have alterations in the intestinal microbial flora. Emerging studies have suggested that pathogenic and non-pathogenic gut bacteria might influence mood-related symptoms and even behavior in animals and humans. In this pilot study, 39 CFS patients were randomized to receive either 24 billion colony forming units of *Lactobacillus casei *strain Shirota (LcS) or a placebo daily for two months. Patients provided stool samples and completed the Beck Depression and Beck Anxiety Inventories before and after the intervention. We found a significant rise in both *Lactobacillus *and *Bifidobacteria *in those taking the LcS, and there was also a significant decrease in anxiety symptoms among those taking the probiotic vs controls (p = 0.01). These results lend further support to the presence of a gut-brain interface, one that may be mediated by microbes that reside or pass through the intestinal tract.

## Background

Chronic Fatigue Syndrome (CFS) is a medically unexplained illness, characterized by persistent and relapsing fatigue [1,2]. This severe pathological fatigue is worsened by periods of physical and mental exertion. Along with the ongoing fatigue, it has also been noted that 97% of CFS patients report neuropsychological disturbances. This can manifest as cognitive dysfunction, sleep disturbances, headaches, and a variety of symptoms in the emotional realm. Of these emotion-related symptoms, anxiety and depression are the most prevalent, with approximately half or patients meeting the criteria for an anxiety disorder or major depressive disorder. Over 40% of patients report symptoms that are often part of anxiety and depressive disorders, including dizziness, lightheadedness, heart palpitations, sleep disturbances, appetite changes and shortness of breath [3].

Many CFS patients also complain of gastrointestinal (GI) disturbances. Indeed, patients with CFS are more likely to report a previous diagnosis of irritable bowel syndrome (IBS), meet diagnostic criteria for IBS and experience IBS related symptoms [4]. While CFS is neither a gastrointestinal nor psychiatric disorder per se, over 50 percent of patients with CFS meet the diagnostic criteria of IBS, and anxiety itself is often a hallmark symptom in those with IBS [5]. Although the mechanisms behind this frequent overlap with IBS are far from understood, some investigators have documented that there are marked alterations in the intestinal microflora of CFS patients, with lower levels of *Bifidobacteria *and higher levels of aerobic bacteria [6].

Recently it was discovered that gut pathogens in the GI tract can communicate with the central nervous system and influence behavior associated with emotion, anxiety in particular, even at extremely low levels and in the absence of an immune response [7,8]. Researchers have also shown that the administration of certain bacteria found in soil may support resilience and positively alter stress-related emotional behavior in animals under experimental stress [9]. In addition, so-called probiotics, or live microorganisms which confer a health benefit on the host, have the potential to influence mood-regulating systemic inflammatory cytokines, decrease oxidative stress and improve nutritional status when orally consumed [6].

This background led some investigators to hypothesize a possible adjunctive therapeutic role of probiotic bacteria in mood-related psychiatric symptoms [10]. Some hints at the utility of probiotics for mood regulation come from a recent human trial involving the administration of *Lactobacillus casei *strain Shirota (LcS) or placebo to 132 otherwise healthy adults. In an intriguing finding, the investigators discovered that those with the lowest scores in the depressed/elated dimension at baseline had significant improvement in mood scores after taking the probiotic compared to the placebo group. The probiotic bacteria and placebo were unable to make a difference in those with the highest baseline mood scores [11]. In addition, ongoing experimental studies in this area have recently shown that in the animal model of depression, the oral administration of a probiotic can increase plasma tryptophan levels, decrease serotonin metabolite concentrations in the frontal cortext and dopamine metabolite concentrations in the amygdaloid cortex [12]. With this background, the current investigation was initiated to determine if orally administered probiotics could make a difference in symptoms of depression and anxiety in adult patients with chronic fatigue syndrome.

### Brief Report

Candidates for inclusion were screened from a pool of CFS patients in a tertiary setting. Those adult patients aged 18–65 meeting the formal diagnostic criteria for CFS, according to published guidelines, [13] were further screened for inclusion based on suitability to complete a two month trial. Excluded were those patients with unstable physical illness and those with a severity of CFS such that they were largely bedridden. Also excluded were patients meeting criteria for psychiatric disorders other than depression and/or anxiety. The study was approved by the institutional review board of the University of Toronto. Patients meeting inclusion criteria provided written, informed consent at the screening visit after the procedures had been fully explained.

CFS patients who met all inclusion/exclusion criteria were evaluated using the Beck Depression Inventory (BDI) and the Beck Anxiety Inventory (BAI). Patients also provided stool samples, taken over 3 days, at the evaluation phase. Kits were provided to the patients according to guidelines of appropriate collection as per the University of Toronto, School of Medicine, Department of Nutritional Sciences. Samples were sent to the Fecal Laboratory of the University of Toronto, Department of Nutrition for evaluation. Using culture technique, the stool samples were assessed for total aerobe, anaerobe, *Lactobacillus *spp, and *Bifidobacteria *spp counts.

After collection of the data and samples at the initial evaluation phase, 39 CFS patients were randomized to begin an intervention phase, an eight-week period where each patient consumed either a specific lactic acid probiotic bacteria or placebo by mouth. After each main meal, or three times daily, patients consumed the contents of an unmarked sachet containing 8 billion colony forming units (cfu) of *Lactobacillus casei *strain Shirota (LcS) or a placebo with identical taste and appearance. Each CFS patient in the active intervention group consumed a total of 24 billion cfu of LcS probiotic per day. Follow-up evaluation phase: After 8 weeks of intervention, LcS or placebo, subjects were re-evaluated by completion of the BDI and BAI, and a second stool analysis was conducted using the same collection and culture methods.

**Figure 1:**
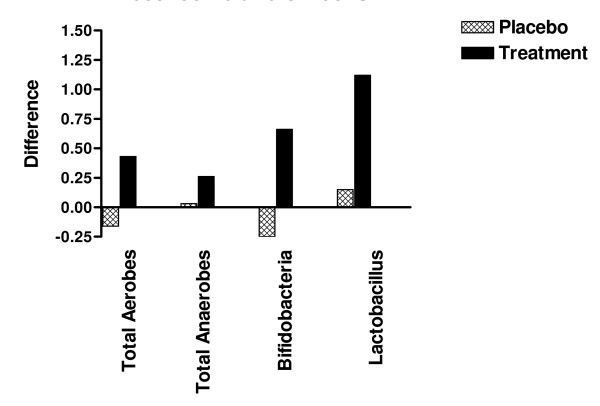
**Difference in fecal bacteria between 0 ands 8 weeks in placebo and treatment groups**.

Thirty five CFS patients, 27 females and 8 males, completed the 8-week investigation. Four patients, two from the LcS probiotic group and two from the placebo group, withdrew from the study for reasons unrelated to the intervention. The LcS probiotic powder was well tolerated and there were no significant adverse events reported in the probiotic or placebo groups. Compared to the placebo control group, the treatment group showed moderate increases in fecal total aerobes and anaerobes and significant increases in fecal total *Bifidobacteria *and *Lactobacillus *(Figure 1). Among the Placebo group only 37.5% of subjects showed an increase in *Bifidobacteria *and 43.8% in *Lactobacillus *compared to 73.7% and 73.7% in the treatment group respectively (Table 1). The number of subjects showing changes in fecal *Bifidobacteria *and *Lactobacillus *is also presented in Table 2. An increase in the number of fecal *Lactobacillus *observed in this study was to be expected since the probiotic sachets contained high levels of a specific strain of this bacteria. The positive results observed with respect to *Bifidobacteria *are very encouraging since *Bifidobacteria *levels have been reported to be low in CFS, and they are generally associated with a healthy colonic environment. [14]. It can therefore be concluded that ingestion of the probiotic capsules contributed towards the predominance of bacteria that are associated with a healthy gastrointestinal system.

**Table 1:** Total Fecal Aerobes, Anaerobes, Bifidobacteria and Lactobacillus in Placebo and Treatment Groups

	**Total Aerobes**			**Total Anaerobes**			**Bifidobacteria**			**Lactobacillus**		
	0 Wk	8 Wk	Dif	0 Wk	8 Wk	Dif	0 Wk	8 Wk	Dif	0 Wk	8 Wk	Dif

Placebo	7.98	7.82	-0.16	9.46	9.49	0.03	6.96	6.60	-0.36	5.81	5.96	0.15

Treatment	7.52	7.95	0.43	9.32	9.58	0.26	6.44	7.10	0.66	6.93	8.05	1.12

Values expressed as mean CFU per gram fresh fecal sample

**Table 2:** Number of Subjects Showing Changes in Fecal Bifidobacteria and Lactobacillus

	**BIFIDOBACTERIA**							**LACTOBACILLUS**						
	
	Total	No change		Increase		Decrease		Total	No change		Increase		Decrease	
	
		No	%	No	%	No	%		No	%	No	%	No	%
Placebo	16	-	-	6	37.5	10	62.5	16	-	-	7	43.8	9	56.2

Treatment	19	-	-	14	73.7	5	26.3	19	1	5.3	14	73.7	4	21.0

When evaluating the BDI and BAI for changes over the course of the 8-week study, we found a statistical difference between the anxiety scores in those taking LcS or the placebo. Overall there was a significant improvement in anxiety among those taking the active LcS compared to the placebo (Table 3). The differences as assessed by the BDI did not reach statistical significance among those taking the active LcS.

**Table 3:** Repeated measures analysis of variance for the BDI and BAI after treatment with placebo or probiotics

	**BDI**			**BAI**		
	df	F	Sig	df	F	sig

**Baseline Placebo vs. Probiotic**	1	1.773	.204	1	.087	.772

**Probiotic Treatment Pre vs. Post**	1	1.197	.292	1	8.415*	.011

**X Interaction**	1	.452	.513	1	.125	.729

*p < 0.05

## Discussion

The idea that implanting the intestines with Lactobacillus strains may improve quality of life and mental health is not a new one. Dr. George Porter Phillips first reported in 1910 that although *Lactobacillus *tablets and powder were ineffective, a gelatin-whey formula with live lactic acid bacteria improved depressive symptoms in adults with melancholia [15]. In a series of case reports, separate researchers concluded in 1923 that 'the administration of acidophilus milk is recommended in the treatment of psychoses as a means to physical betterment' [16]. In this pilot study we found that the oral administration of *Lactobacillus casei *strain Shirota (LcS, Yakult Honsha, Tokyo, Japan) caused a significant rise in fecal *Bifidobacteria spp*. and *Lactobacillus spp*. The rise in *Lactobacilli *was an expected finding, although the concomitant rise in *Bifidobacteria *suggests that there may be far reaching effects of oral probiotics on other microbial residents of the gastrointestinal tract. This finding supports previous research showing that the oral administration of *Lactobacillus plantarum *299 V caused a significant rise in fecal *Bifidobacteria *levels [17]. In this case the elevation of *Bifidobacteria *levels should be considered a positive finding, particularly when considering that *Bifidobacteria *levels may be low in CFS. Also of relevance is a recent experimental study which has shown that a specific strain of *Bifidobacteria *can boost plasma tryptophan levels and alter serotonin and dopamine turnover in areas of the brain associated with depression and anxiety [12]. We also found a significant reduction in anxiety scores among those CFS patients consuming the LcS bacteria. The group differences in anxiety are noteworthy since anxiety is a frequent mental health symptom reported by CFS patients.

In recent years the interface between neuropsychiatry and gastroenterology has converged into a new discipline referred to as enteric neuroscience. Emerging studies have shown that intestinal bacteria may directly communicate with the central nervous system by way of the vagal sensory nerve fibers and the peripheral immune system. Indeed, experimental studies have shown that even minute doses of microbes within the gastrointestinal tract, levels that do not trigger an immune response, are capable of influencing neurotransmission in the paraventricular hypothalamus, the central nucleus of the amygdala, and the bed nucleus of the stria terminalis [8]. All three of these regions are involved in the processing of emotions related to anxiety and mood. It is also true that quantitative alterations in the make-up of gastrointestinal microbes are a consequence of states of stress and fear, and alterations in the gut microflora have recently been associated with impaired glucose control and obesity [18,19].

More hints of a connection between intestinal microflora and brain function come from studies in the autistic spectrum. Research has shown marked alterations of the gastrointestinal microflora in autism, with specific elevations in various *Clostridium *spp. [20]. Some researchers speculate that low-grade chronic intestinal inflammation induced by elevations in bacteria such as potentially pathogenic *Clostridium *spp may be directly influencing brain centers. Experimental studies show that indeed chronic gut inflammation leads to activation of areas of the brain associated with mental health and behavioral disorders, including the hypothalamus, amygdala and cortical centers [21]. While we did not look specifically at *Clostridium *spp. in this pilot investigation, it has been noted that *Lactobacillus *can competitively displace *Clostridium *and other potentially pathogenic gut bacteria [22]. Propionic acid is a short chain fatty acid produced primarily by *Clostridium *and *Bacteroides *spp.; emerging research suggests that this acid may be involved in anxiety. Elevated production of propionic acid in the gut has been shown to increase behaviors associated with anxiety and aggression in animals [23]. It has also been shown recently that when propionic acid gains access to the brain it can impair the social behavior of animals. Changes to the animal behavior include decreased playful behavior, increasing social isolation, and an increase in repetitive behaviors that may indicate anxiety [24]. While human data is lacking, a study in animals did show that the LcS as used in our study can lower cecal propionate levels [25].

Some researchers have stated that the so-called 'hygiene hypothesis' extends into the realm of mental health disorders as well. The hygiene hypothesis is the proposition that the documented rise in chronic inflammatory disorders (allergies, autoimmunity, and inflammatory bowel disease) within developed countries is driven by a changing microbial environment, an absence of beneficial bacteria that has in turn altered the immuno-regulatory circuits which normally keep inflammatory responses in check [26]. Many mental health conditions, and so-called functional somatic disorders such as CFS, have been well-documented to have elevations in inflammatory cytokines, and these inflammatory cytokines at even low levels can produce symptoms of anxiety and depression in otherwise healthy adults [26]. Therefore, since orally administered probiotics can decrease inflammatory cytokines in humans, it has been postulated that bacteria may be used to positively influence mood in patient populations where both emotional symptoms and inflammatory immune chemicals are elevated [10]. It is becoming increasingly clear that anxiety and stress itself may lower levels of fecal lactic acid bacteria, and this, in turn, may compromise various aspects of health [27].

Overall the results suggest that specific strains of probiotic bacteria may have a role to play in mediating some of the emotional symptoms of CFS and other related conditions. However, it is important to note that this is a small pilot study and broad conclusions cannot be drawn at this time. Since we did not evaluate bowel function during the study, it is entirely possible that the decreased anxiety was a consequence of improved bowel function. In an unexplained medical condition such as CFS, where over 70% of patients meet the criteria for IBS, it is possible that regulation of bowel movements made a difference in mental state. Indeed LcS has been shown to regulate bowel function and decrease constipation in a controlled trial [28]. It is also true that LcS has been shown to reduce small intestinal bacterial overgrowth and the subjective reporting of the passage of gas in patients with IBS [29]. This is of significance because SIBO and intestinal permeability often overlap, and patients with chronic fatigue syndrome are known to have both increased intestinal permeability and SIBO. Indeed, correction of SIBO and intestinal permeability has been shown to improve symptoms in CFS and depressive disorders [30,31]. Therefore, it is entirely possible that our results are an artifact of improved gut structure and function via the LcS restoration of a healthy intestinal biofilm. However, a recent study using the same LcS strain in healthy adults suggests that there may be a more direct microbial influence on emotional state. In healthy adults who were reported to be more depressed/less elated in daily functioning at baseline, there was significant improvement in mood scores after taking the probiotic. In that controlled trial the improvements in mood were not related to changes in bowel function [11].

This preliminary research raises many questions regarding possible mechanisms whereby probiotics might influence anxiety and depression. The results of the present study should be viewed simply as a stimulus for further research. Follow-up studies with probiotics should further examine specific gut microbes, intestinal structure and function as well as physiological markers associated with anxiety and depression. These may include inflammatory cytokines and other immune chemicals, blood tryptophan levels and urinary metabolites of neurotransmitters.

## Competing interests

This pilot study was funded by Yakult Honsha, Tokyo, Japan. None of the authors have any financial relationship with the funding body, Yakult Honsha, and have no interest in the sales of this product under investigation.

## Authors' contributions

AVR coordinated the study, completed the stool analysis and data analysis, ACB and TMB performed the patient recruitment, screening, sample and data collection, MK, CI and JMB collected and evaluated the BDI and BAI questionnaires and completed the data analysis, ACL designed the investigation and drafted the manuscript. All authors reviewed the data and content, assisted in the final manuscript construction and agree to its content.

## References

[B1] Komaroff AL, Buchwald D (1991). Symptoms and signs of chronic fatigue syndrome. Rev Infect Dis.

[B2] Komaroff AL, Fagioli LR, Geiger AM, Doolitle TH, Lee J, Kornish RJ (1996). An examination of the working case definition of chronic fatigue syndrome. American J Med.

[B3] Wessely S, Chalder T, Hirsch S, Wallace P, Wright D (1996). Psychological symptoms, somatic symptoms, and psychiatric disorder in chronic fatigue and chronic fatigue syndrome: a prospective study in the primary care setting. Am J Psychiatry.

[B4] Aaron LA, Burke MM, Buchwald D (2000). Overlapping conditions among patients with chronic fatigue syndrome, fibromyalgia and temporomandibular disorder. Arch Intern Med.

[B5] Whitehead WE, Palsson O, Jones KR (2002). Systematic review of the comorbidity of irritable bowel syndrome with other disorders: what are the causes and implications?. Gastroenterology.

[B6] Logan A, Rao V, Irani D (2003). Chronic fatigue syndrome: lactic acid bacteria may be of therapeutic value. Med Hypotheses.

[B7] Lyte M, Varcoe JJ, Bailey MT (1998). Anxiogenic effect of subclinical bacterial infection in mice in the absence of overt immune activation. Physiol Behav.

[B8] Goehler LF, Lyte M, Gaykema RP (2007). Infection-induced viscerosensory signals from the gut enhance anxiety: implications for psychoneuroimmunology. Brain Behav Immun.

[B9] Lowry CA, Hollis JH, De Vries A, Pan B, Brunet LR, Hunt JR (2007). Identification of an immune-responsive mesolimbocortical serotonergic system: potential role in regulation of emotional behavior. Neuroscience.

[B10] Logan A, Katzman M (2005). Major depressive disorder: probiotics may be an adjuvant therapy. Med Hypotheses.

[B11] Benton D, Williams C, Brown A (2007). Impact of consuming a milk drink containing a probiotic on mood and cognition. Eur J Clin Nutr.

[B12] Desbonnet L, Garrett L, Clarke G, Bienenstock J, Dinan T (2008). The probiotic *Bifidobacteria Infantis*: an assessment of potential antidepressant properties in the rat. J Psychiatr Res.

[B13] Carruthers BM, Jain AK, De Meirleir KL, Peterson DL, Klimas NG, Lerner AM (2003). Myalgic encephalomyelitis/chronic fatigue syndrome: Clinical working case definition, diagnostic and treatment protocols. J Chronic Fatigue Syndrome.

[B14] Mitsuoka T (1992). Intestinal flora and aging. Nutr Rev.

[B15] Phillips JGP (1910). The treatment of melancholia by the lactic acid bacillus. J Mental Sci.

[B16] Julianelle LA, Ebaugh FG (1923). Implantation of Bacillus Acidophilus in perswons with psychoses. Arch Neurol Psychiatr.

[B17] Johansson ML, Nobaek S, Berggren A, Nyman M, Bjorck I, Ahrne S (1998). Survival of Lactobacillus plantarum DSM 9843 (299 v), and effect on the short-chain fatty acid content of faeces after ingestion of a rose-hip drink with fermented oats. Int J Food Microbiol.

[B18] Bailey MT, Engler H, Sheridan JF (2006). Stress induces the translocation of cutaneous and gastrointestinal microflora to secondary lymphoid organs of C57BL/6 mice. J Neuroimmunol.

[B19] Cani PD, Delzenne NM (2007). Gut microflora as a target for energy and metabolic homeostasis. Curr Opin Clin Nutr Metab Care.

[B20] Parracho HM, Bingham MO, Gibson GR, McCartney AL (2005). Differences between the gut microflora of children with autistic spectrum disorders and that of healthy children. J Med Microbiol.

[B21] Welch MG, Welch-Horan TB, Anwar M, Anwar N, Ludwig RJ, Ruggiero DA (2005). Brain effects of chronic IBD in areas abnormal in autism and treatment by single neuropeptides secretin and oxytocin. J Mol Neurosci.

[B22] Ramiah K, van Reenen CA, Dicks LM (2008). Surface-bound proteins of Lactobacillus plantarum 423 that contribute to adhesion of Caco-2 cells and their role in competitive exclusion and displacement of Clostridium sporogenes and Enterococcus faecalis. Res Microbiol.

[B23] Hanstock TL, Clayton EH, Li KM, Mallet PE (2004). Anxiety and aggression associated with the fermentation of carbohydrates in the hindguts of rats. Physiol Behav.

[B24] Shultz SR, MacFabe DF, Ossenkopp KP, Scratch S, Whelan J, Taylor R, Cain DP (2008). Intercerebroventricular injection of propionic acid, an enteric bacterial metabolic end-product, impairs social behavior in the rat: implications for the animal model of autism. Neuropharmacology.

[B25] Ohashi Y, Tokunaga M, Ushida K (2004). The effect of Lactobacillus casei strain Shirota on the cecal fermentation pattern depends on the individual cecal microflora in pigs. J Nutr Sci Vitaminol (Tokyo).

[B26] Rook GA, Lowry CA (2008). The hygiene hypothesis and psychiatric disorders. Trends Immunol.

[B27] Knowles SR, Nelson EA, Palombo EA (2008). Investigating the role of perceived stress on bacterial flora and salivary cortisol secretion: a possible mechanism underlying susceptibility to illness. Biol Psychol.

[B28] Koebnick C, Wagner I, Leitzman P, Stern U, Zunft HJ (2003). Probiotic beverage containing Lactobacillus casei Shirota improves gastrointestinal symptoms in patients with chronic constipation. Can J Gastroenterol.

[B29] Barrett JS, Canale K, Gearry RB, Irving PM, Gibson PR (2008). Probiotic effects on intestinal fermentation patterns in patients with irritable bowel syndrome. World J Gastroenterol.

[B30] Pimentel M, Hallegua D, Chow EJ, Wallace D, Bonorris G, Lin HC (2000). Eradication of small intestinal bacterial overgrowth decreases symptoms in chronic fatigue syndrome: a double blind, randomized study. Gastroenterology.

[B31] Maes M, Leunis JC (2008). Normalization of leaky gut in chronic fatigue syndrome (CFS) is accompanied by a clinical improvement: effects of age, duration of illness and the translocation of LPS from gram-negative bacteria. Neuro Endocrinol Lett.

